# Healthy lifestyle as predictors of common mental disorder during coronavirus disease

**DOI:** 10.1590/1806-9282.20231004

**Published:** 2024-01-26

**Authors:** Laianne Liliane Pereira Troncha de Castro, Henrique Porcatti Walsh, Marilita Falangola Accioly, Lislei Jorge Patrizzi Martins, Ana Carolina Otoni Oliveira, Lívia Pires Marra Graffitti, Maycon Souza Pegorari, Isabel Aparecida Porcatti de Walsh

**Affiliations:** 1 Universidade Federal do Triângulo Mineiro – Uberaba (MG), Brazil.; 2 Medicine, Padre Albino Foundation – Catanduva (SP), Brazil.; 3 Universidade Federal do Triângulo Mineiro, Postgraduate Program in Physiotherapy – Uberaba (MG), Brazil.

**Keywords:** COVID-19, Healthy lifestyle, Mental health

## Abstract

**OBJECTIVE:**

The objective of the present study was to verify the indication of common mental disorder and changes in healthy lifestyle among individuals affected by coronavirus disease, as well as to evaluate if changes in healthy lifestyle are predictors of common mental disorder.

**METHODS:**

This descriptive, cross-sectional study employed an exploratory approach and quantitative methodology, using the Self-Reporting Questionnaire to assess the indication of common mental disorder and questions regarding healthy lifestyle during the pandemic.

**RESULTS:**

A total of 280 individuals affected by coronavirus disease, aged 18 years and above, participated in the study. The average indication for common mental disorder was 5.0±5.34. The average age was characterized by adults (41.24±14.03 years), with the majority being women (57.9%), White (51.4%), and those in stable relationships (55.7%). Worsening sleep quality (β==6.327; p<0.001) was the main predictor of common mental disorder, followed by female gender (β==2.814; p<0.001) and worsening dietary habits (β==2.227; p<0.012).

**CONCLUSION:**

These factors should be considered in the assessment of individuals affected by coronavirus disease to provide comprehensive care.

## INTRODUCTION

The scenario of isolation, uncertainties, negative news from the media, and fear of falling ill, along with the economic, social, and environmental impacts caused by the coronavirus, has led to mental disorders^1^, with anxiety and depression being common reactions, especially among those who were hospitalized due to concerns about their own health or that of others, the need for physical isolation, the potential risk of death, concerns about infecting others, or leaving family members who require care alone^2^. In this sense, a meta-analysis identified mental health disorders in affected populations, with prevalences of depression (15.97%), anxiety (15.15%), insomnia (23.87%), psychological stress (13.29%), and post-traumatic stress disorder (21.94%)^3^.

Common mental disorder (CMD) is defined as a set of somatic, anxious, and depressive manifestations, such as memory and concentration difficulties, irritability, insomnia, fatigue, and feelings of worthlessness, affecting cognitive, physical, emotional, and behavioral functions^1^. It is a major public health problem as it increases the demand for and costs of healthcare services, directly interfering with the quality of life of individuals and their families^4^.

However, adopting a healthy lifestyle, such as regular physical activity, a balanced diet, good sleep quality, and smoking and alcohol control, as well as family support and a good assessment of quality of life are essential for maintaining good mental health. However, during the coronavirus disease 2019 (COVID-19) pandemic, there has been a worsening of lifestyle habits, with an increase in behaviors that pose a risk to health^5^.

Considering the recommendation of the Pan American Health Organization (PAHO)^6^ regarding the provision of basic psychosocial and mental health support to those affected by COVID-19, questioning their needs, concerns, and conducting immediate assessments of anxiety, depressive symptoms, along with psychosocial support strategies and management of sleep-related problems, as well as the importance of promoting healthy lifestyle for good physical and mental health, identifying possible relationships between changes in healthy lifestyle and CMD in individuals affected by COVID-19 can provide support to the healthcare system in addressing these issues, creating health education programs and activities that enable the restoration of healthy habits, considering comprehensive care.

The objective of the present study was to verify the indication of CMD and changes in healthy lifestyle among individuals affected by COVID-19, as well as to evaluate if changes in healthy lifestyle are predictors of CMD.

## METHODS

### Study design and sample

This was a descriptive, cross-sectional study with an exploratory approach and quantitative methodology, approved by the Research Ethics Committee of the Federal University of Triângulo Mineiro (UFTM) under number 4647292.

Men and women aged 18 years or older who were affected by COVID-19 in a city in the interior of Minas Gerais, Brazil, participated in the study. The municipal health department provided the researchers with a list of notified individuals from March 1, 2020, to July 27, 2021, including personal data such as name, date of birth, and telephone number. Out of a total of 31,123 individuals aged 18 years and above, the sample size was calculated using the formula for simple proportion and finite population, with a margin of error of 10% and a confidence level of 95%, indicating a sample size of 201 affected individuals. A random selection was then performed to choose the participants.

Contact was made by telephone, following a standardized script and terms to be used in the approach, with three attempts made to reach each selected individual. During the call, when the participant agreed to participate in the research, he or she was informed that the call would be recorded. In the second step, if the participant did not want to answer by phone, he or she was invited to respond through a link provided via WhatsApp or email.

The participants were made aware of the Free and Informed Consent Form (FICF), and if they agreed to participate, they verbally expressed their agreement during the phone call. If they chose to respond directly through the provided link via WhatsApp or email, they downloaded the FICF and marked their acceptance on the form to proceed with the questionnaire.

Finally, participants were provided with an explanatory and illustrated booklet via WhatsApp or email, containing information on the topic, following recommendations from the World Health Organization (WHO) and the Ministry of Health.

**Figure 1:**
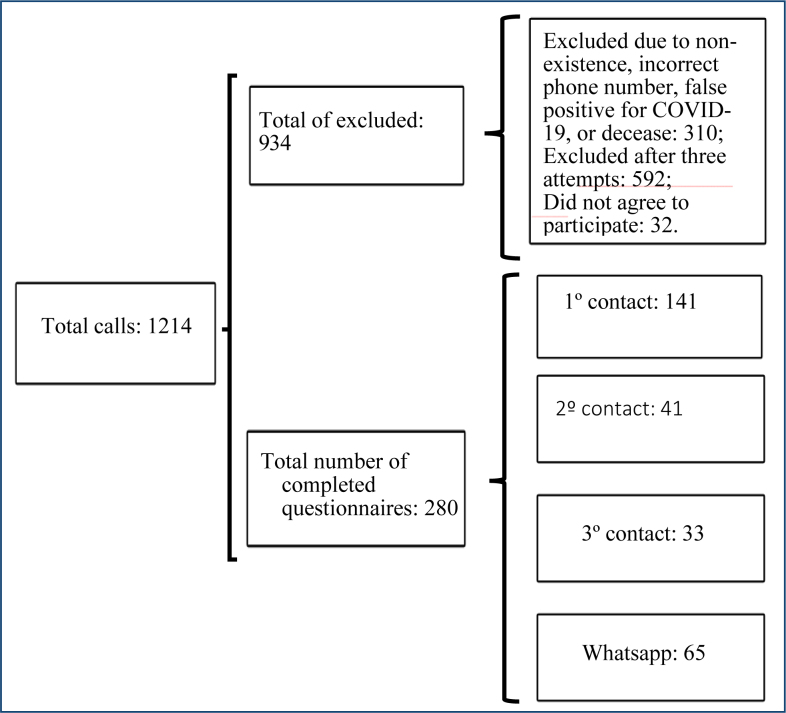
Flowchart of study participants.

Source: Researchers' data, 2022. Minas Gerais, Brazil.

During the data collection period (August 2021 to January 2022), a total of 1,214 calls were made. Out of these, 902 individuals were excluded, 32 declined to participate in the research, and 280 agreed to participate by phone or via WhatsApp, as represented in the flowchart in Figure 1.

Inclusion criteria were individuals diagnosed with COVID-19, registered by the municipality's health department, aged 18 years or above, who agreed to participate in the research after completing the FICF. Exclusion criteria were incomplete evaluation instruments and hospitalized or institutionalized individuals.

### Variables and measures

Regarding sociodemographic aspects, age, gender, race, and marital status were considered. To assess the suspicion (presence or absence) of CMD, the Self-Reporting Questionnaire (SRQ-20) was used. This instrument, developed by the WHO for detecting symptoms and suggesting the level of suspicion (presence or absence) of minor mental disorders such as depression, anxiety, and stress, was adapted for national studies by Santos et al.^7^ and is considered an easily applicable instrument, with the obtained scores related to the probability of CMD ranging from 0 (no probability) to 20 (extreme probability).

Changes in healthy lifestyle were assessed based on responses to questions about improvements, no changes, or worsening of physical activity, diet, smoking, alcohol consumption, and sleep during the pandemic.

### Data analysis

Descriptive statistical analysis was performed, including absolute and percentage frequencies, as well as means and standard deviations. The Mann-Whitney test was used to verify the associations between CMD and sociodemographic characteristics and changes in lifestyle habits, with a significance set at p<0.05. Linear regression analysis was conducted to evaluate the predictors of CMD using a 95% confidence interval (CI) and a significance level of 5%. The minimum prerequisites of normality, linearity, and homoscedasticity of residuals, as well as the absence of multicollinearity, were considered.

## RESULTS

A total of 280 individuals affected by COVID-19, aged 18 years and above, participated in the study. The average indication for CMD was 5.0±5.34. The average age was characterized by adults (41.24±14.03 years), with the majority being women (57.9%), White (51.4%), and those in stable relationships (55.7%).

Being female and reporting worsening of physical activity, sleep quality, diet, and alcohol consumption were associated with higher average indications for the development of CMD (p<0.05), as presented in Table 1.

**Table 1:** Sample characteristics and distribution of means and proportions in groups according to the indication of developing a common mental disorder among individuals affected by coronavirus disease.

Variables		TMC code Mean±SD	p-value	Full sample n=280
Age (years)			0.715	
Sex				
	Feminine	6.69±5.75	<0.001*	162 (57.9)
	Masculine	2.69±3.65		118 (42.1)
Color				
	White	4.79±5.31	0.396	144 (51.4)
	Afrodescendant	5.23±5.38		136 (48.6)
Marital status				
	Without union	5.23±5.18	0328	124 (44.3)
	In union	4.83±5.48		156 (55.7)
Practice of physical activity				
	Improved	4.05±4.82	<0.001*	43 (15.4)
	Has not changed	3.45±4.24		129 (46.1)
	Got worse	7.24±5.95		108 (38.6)
Food				
	Improved	4.64±5.36	<0.001*	42 (15)
	Has not changed	3.73±4.29		183 (65.4)
	Got worse	9.53±5.08		55 (19.6)
Smoking				
	Improved	6.83±5.42	0.056	6 (2.1)
	Has not changed	4.78±5.19		263 (93.9)
	Got worse	9.27±7.27		11 (3.9)
Alcoholism				
	Improved	6.91±5.17	0.016*	22 (7.9)
	Has not changed	4.60±5.06		243 (76.8)
	Got worse	8.67±7.93		15 (5.4)
Sleep				
	Improved	2.07±2.76	<0.001*	15 (5.4)
	Has not changed	2.69±3.78		161 (57.5)
	Got worse	9.01±9.32		104 (37.1)

CMD: common mental disorder; n: number of subjects; mean±standard deviation

* p<0.05, Mann-Whitney test. Source: Survey data. Uberaba-MG, 2022, n=280

Multivariate analysis indicated that worsening sleep quality (β==0.404; p<0.001) was the main predictor of CMD, followed by female gender (β=-0.311; p<0.001), worsening diet (β==0.172; p=0.002), and worsening physical activity (β==0.112; p=0.044) (Table 2).

**Table 2:** Common mental disorder and changes in lifestyle in people affected by coronavirus disease.

	TMC code					
	β	Standard error	T	p-value	Lower limits	Upper limits
Practice of physical activity	0.112	0.433	2.026	0.044*	0.024	1.731
Food	0.172	0.523	3.085	0.002*	0.584	2.647
Alcoholism	-0.033	0.772	-0.625	0.533	-2.004	1.039
Sleep	0.404	0.532	7.285	<0.001*	2.828	4.926
Sex	-0.311	0.574	-5.952	<0.001*	-4.544	-2.283

**β**: standardized coefficient; T: *t*-test; CI: confidence interval.

* p<0.05. Source: Survey data. Uberaba-MG, 2022, n=280.

## DISCUSSION

This study identified that worsening sleep quality was the main predictor of CMD, followed by female gender, worsening diet, and worsening physical activity.

During social isolation, there has been a significant impact on mental health, with poor habits, including an unbalanced diet, combined with the use of alcohol and tobacco, leading to sleep disorders and the onset of mental health problems^8^. Both the quantity and quality of sleep have been affected^9^. It is known that sleep is essential for human development and well-being. Thus, worsened sleep during the pandemic may also be associated with the development of other disorders.

Regarding diet, it should be considered that mental disorders are caused by failures in the communication of neurotransmitters with the nervous system, which affect psychomotor activities, appetite, sleep, and mood. Serotonin and dopamine are the main neurotransmitters associated with depression^10^, and nutrition contributes to the production of these neurotransmitters, with nutrients serving as raw materials and regulating their quantities in the body. Vitamins, amino acids, and minerals are the most prominent nutrients in this process^11^. Additionally, stress triggers increased food consumption, particularly comfort foods, which can lead to significant changes in sleep and drive reward-seeking behaviors that increase the chances of uncontrolled eating^12^.

As for female gender being a predictor of CMD, many studies have highlighted that women are more affected by the onset of mental disorders^13–16^. Being female has been associated with worsened mental health, especially in individuals affected by the virus when compared to those who were not infected^17^. In patients evaluated 3 months after the acute phase of the disease, it was noted that individuals with a psychiatric history and females may exhibit greater symptoms for the development of depression^18^. It is worth considering that Brazil already had one of the highest rates of mental health problems in the world even before the pandemic^19^. During the pandemic, women were found to have three times higher chances of developing mental disorders compared to men, as demonstrated by a study conducted in Rio Grande do Sul State^20^. Thus, appropriate attention should be given to this segment of the population.

One limitation of our study was its cross-sectional design, which limited our ability to establish a causal relationship between CMD and lifestyle habits. As a strength, we consider that it was conducted on a representative sample of the population affected by COVID-19 in the municipality.

Considering the need to assess the consequences of mental health in the care of clinical conditions, further studies should consider the importance of evaluating changes in healthy lifestyle in the population and their consequences for mental health. This would help establish public policies with measures focused on the mental health of those affected and those involved in primary healthcare and specialized outpatient care.

## CONCLUSION

Worsened sleep quality, diet, physical activity, and being female were predictors for the development of CMD. These factors should be considered in the assessment of individuals affected by COVID-19 for comprehensive care.
